# Food Insecurity, Burnout, and Social Isolation Among Resident and Fellow Physicians

**DOI:** 10.1001/jamanetworkopen.2025.50044

**Published:** 2025-12-17

**Authors:** Larissa R. Thomas, Liselotte N. Dyrbye, Daniel Satele, Saadia Akhtar, Jonathan A. Ripp, Katherine A. Julian, Diane Sliwka, Colin P. West

**Affiliations:** 1 Department of Medicine, University of California, San Francisco School of Medicine, San Francisco; 2 Department of Medicine, University of Colorado Anschutz School of Medicine, Aurora; 3 Department of Quantitative Health Sciences, Mayo Clinic School of Medicine, Rochester, Minnesota; 4 Department of Emergency Medicine, Icahn School of Medicine at Mount Sinai, New York, New York; 5 Office of the Dean, Icahn School of Medicine at Mount Sinai, New York, New York; 6 Department of Medicine, Mayo Clinic School of Medicine, Rochester, Minnesota

## Abstract

**Importance:**

Little is known about food insecurity (FI) among graduate medical education (GME) trainees or how FI might relate to well-being outcomes in this population.

**Objectives:**

To assess the prevalence of and factors associated with FI among GME trainees and to investigate the associations of FI with well-being outcomes such as burnout and social isolation in this population.

**Design, Setting, and Participants:**

In this cross-sectional study, a survey was distributed among 3408 resident and fellow physicians at 4 geographically distinct sites within 2 US academic institutions from May 2 to June 21, 2023. All resident and fellow physicians appointed at both institutions were eligible and were invited by email to participate.

**Main Outcomes and Measures:**

The primary outcome was prevalence of FI. Measurement instruments included a 2-item FI screening tool, a 2-item measure of burnout, items addressing intent to stay at one’s institution, social isolation, and demographic characteristics. Univariable analysis and multivariable Poisson and linear regression were used to characterize prevalence and factors associated with FI and associations with well-being outcomes and intent to stay at the institution.

**Results:**

Of those eligible, 1656 residents and fellows participated in the survey (a response rate of 48.6%). Of these respondents, 735 of 1458 (50.4%) who indicated their gender identified as men and 365 of 1402 (26.0%) who indicated their parental status reported having children. Of the 1551 respondents who reported their age, 519 (33.5%) were younger than 30 years, 770 (49.6%) were aged 31 to 35 years, 169 (10.9%) were aged 36 to 40 years, and 53 (3.4%) were older than 40 years. Among 1457 respondents indicating their race and ethnicity, the majority were Asian (310 [21.3%]) or White (654 [44.9%]). The overall prevalence of FI was 13.7%. FI proportions differed across training sites, with higher levels in large metropolitan locations (15.6%, 17.0%, and 21.3% for the 3 sites in large metropolitan areas vs 4.5% for the site in a small metropolitan area; *P* < .001). There were also differences by postgraduate year (16.9% for postgraduate year 1 vs 10.1% for postgraduate year ≥5; *P* = .003) and race and ethnicity (22.4% for Black or African American trainees compared with 8.4% for White trainees; *P* = .04). Those with FI were more likely to experience burnout (adjusted relative risk, 1.37 [95% CI, 1.18-1.60]; *P* < .001), were less likely to consider remaining at their institution after training (adjusted relative risk, 0.81 [95% CI, 0.68-0.98]; *P* = .02), and had higher social isolation scores (T-score parameter estimate 2.37 [95% CI, 0.89-3.86]; *P* = .002).

**Conclusions and Relevance:**

In this cross-sectional study, nearly 1 in 7 GME trainees screened positive for FI. FI was associated with important well-being outcomes including burnout. These findings suggest that academic medical institutions should pursue systemic solutions to address FI among resident and fellow physicians as a means of supporting their well-being.

## Key Points

**Question:**

What are the prevalence of and factors associated with food insecurity (FI) among graduate medical education trainees, and what is the association between FI and well-being outcomes such as burnout and social isolation?

**Findings:**

In this cross-sectional study of 1656 resident and fellow physicians at 4 geographically distinct training sites within 2 large academic medical institutions in the US, the prevalence of FI was 14%, with significant differences by race and ethnicity, year of training, and site. Those with FI were more likely to experience burnout.

**Meaning:**

These findings suggest that addressing FI in graduate medical education trainees is an important means of supporting their well-being.

## Teaser

This cross-sectional study assesses the prevalence of and factors associated with food insecurity among graduate medical education trainees and investigates the associations between food insecurity and well-being outcomes such as burnout and social isolation.

## Introduction

Food insecurity (FI) is defined as having uncertain access to food or not having enough food at some point in the last year.^1^ In 2023, 13.5% of households in the US met criteria for FI, representing approximately 47.4 million individuals.^2^ FI is a complex and multifaceted challenge influenced by social, geographic, and structural factors. A major driver for FI is a mismatch between available income and cost of living, which may be due to low wages, or wages that are low relative to the local cost of living.^3^ Additionally, those living in densely populated urban areas have a higher prevalence of FI than those living in suburban areas.^2^ Populations who disproportionately experience FI include low-income families with children, racial and ethnic minority individuals, people with disabilities, and caregivers.^2,3^ Racial and ethnic minority individuals may also have a higher risk for FI independent of their economic status due to historic barriers to education, employment, or housing opportunities.^4,5^ FI is less prevalent in households that include married couples with children.^2^ Prior studies have also found a high prevalence of FI among student and learner populations, including undergraduates, graduate students, and nonphysician postdoctoral trainees.^6,7^ Recent studies have reported that up to 25% of medical students experience FI,^8,9^ with higher rates in 1 study among those with a racial or ethnic identity that is underrepresented in medicine (defined as American Indian or Alaska Native, Black, or Hispanic or Latino), those from low-income families, and those with a disability.^9^

FI is recognized as a major health indicator, as people who screen positive for FI are more likely to experience stigma, social isolation, and a variety of other negative physical and mental health consequences.^7,10,11,12^ Medical students experiencing FI have reported experiencing guilt, social isolation, and decreased physical activity.^13^ Since those experiencing FI may be unlikely to self-identify or seek resources due to stigma,^13,14,15^ awareness of which groups may be at risk for FI may help to guide measures to mitigate this risk.

To date, risk for FI has not been characterized among residents and clinical fellows in graduate medical education (GME). While practicing physicians are likely to be at low risk for FI given that their incomes are typically in the top tier of income categories, residents and fellows may be at higher risk for FI due to their lower salaries, especially if a high proportion of their salary must be allocated for housing costs and other expenses such as student loans.^16,17,18^ Based on studies in medical students, financial strain, which is associated with risk for FI, may be especially high for residents who do not have a financial safety net or generational wealth, such as those who are the first in their family to attend college.^19,20^ Previous studies have also shown associations between high debt burden and burnout.^21,22^ Burnout affects roughly 50% of trainees nationally,^23^ and debt burden among medical trainees has increased in recent years.^24^ This multisite study therefore sought to characterize the risk of FI in residents and fellows and investigate associations with burnout and social isolation, both of which are important contributors to well-being. Additionally, given previously described associations between well-being outcomes and retention at an institution,^25,26^ we hypothesized that trainees experiencing FI may be less likely to consider staying at their institution for further training or a faculty position.

## Methods

This cross-sectional study was reviewed by each institution’s institutional review board and was determined to be exempt research under Revised Common Rule Category 2 because it only included survey procedures; written consent was waived to protect anonymity. Participants received an electronic information sheet with the invitation to participate and indicated consent to participate when they took the survey. The study followed the Strengthening the Reporting of Observational Studies in Epidemiology (STROBE) reporting guideline.

### Study Sample

 We distributed a survey to 3408 resident and fellow physicians at 4 geographically distinct training sites within 2 academic medical institutions in 4 US states between May 2 and June 21, 2023. Three sites were classified as being in a large central metropolitan area (population 1 million or more) and 1 site was classified as being in a small metropolitan area (population 50 000-249 000) according to the National Center for Health Statistics.^27^ All resident and fellow physicians at each institution were eligible to take the survey. The anonymous survey was sent electronically with follow- up email reminders and did not retain any identifiers.

### Survey Instrument

The survey instrument included self-reported demographic questions regarding age, gender identity, race and ethnicity (categorized as Asian, Black or African American, Hispanic or Latino, Middle Eastern or North African, White, other race or ethnicity [including American Indian or Alaska Native, Native Hawaiian or Other Pacific Islander, and multiracial], and prefer not to answer), sexual orientation, parental status, specialty, and postgraduate year. It also included validated tools for FI, social isolation, and burnout and questions regarding intention to stay for further training or for a faculty position at their site. Race and ethnicity data were collected because of established associations with FI and multiple dimensions of well-being. All responses were optional.

#### FI

Risk for FI was measured using a 2-item screening measure based on the US Household Food Security Survey Module (HFSSM) that has been widely applied to screen for risk of FI.^28^ A version of these questions has been disseminated as the Hunger Vital Sign.^29^ These questions have been validated in children and adults and in health care settings. Compared with the 18-item, gold standard HFSSM, this 2-item screening tool has a sensitivity of greater than 97% and a specificity of 93% when including households at all income levels.^28^ The 2 items are: “We worried whether our food would run out before we got money to buy more” and “The food we bought just didn’t last, and we didn’t have money to get more.” For each item, respondents are asked to select whether the statement was often true, sometimes true, or never true for their household in the last 12 months. Respondents are considered to screen positive for FI if they answer that the statement is often true or sometimes true vs never true for either or both of the items.^28,29^

#### Social Isolation

The 4-item Patient Reported Outcomes Measurement Information System (PROMIS) Social Isolation scale, which can be used both in the general population and for individuals living with chronic conditions, was used to assess social isolation.^30^ This measure assesses perceptions of being avoided, excluded, detached, and disconnected from or unknown by others. Responses are on a 5-point scale ranging from 1 (never) to 5 (always), and total scores range from 4 to 20, with higher scores indicating greater social isolation. The summed score is then converted to a nationally normed T-score on a scale of 0 to 100, with each 10-point interval equating to 1 SD.

#### Burnout

Burnout was assessed using 1 item from each of the emotional exhaustion and depersonalization domains of the Maslach Burnout Inventory, previously demonstrated to correlate strongly with the respective full Maslach Burnout Inventory domains,^31,32^ each with answer choices on a 7-point ordinal scale. This instrument has also been dichotomized as a measure of burnout prevalence, with a response of “once a week or more” on one or both items indicating overall burnout. These items were used under license with Mind Garden Inc.

### Statistical Analysis

Standard descriptive statistics were calculated and differences in factors between those with and without a positive FI screen were evaluated using χ^2^ tests. Multivariable modified Poisson regression with robust error variances was performed with positive FI screen as the dependent factor, with the aforementioned demographic factors as independent variables. Only respondents with values for all independent variables were included in the multivariable model (n = 1334). To examine the association of FI with overall burnout and intent to stay at the institution, multivariable modified Poisson regression was again performed separately for each dependent variable. To examine the association of FI with social isolation, multiple linear regression was performed using the T-score for the PROMIS Social Isolation scale as the dependent factor. The threshold for statistical significance was *P* < .05, and all analyses were performed using SAS, version 9.4 (SAS Institute Inc).

## Results

### Participants

Of 3408 eligible residents and fellows, 1656 participated in the survey (response rate of 48.6% across all 4 sites [range, 46.8%-50.9%]). Among 1458 respondents indicating their gender, 735 (50.4%) were men, 602 (41.3%) were women, 12 (0.8%) were another gender, and 109 (7.5%) preferred not to answer. Of the 1551 respondents who reported their age, 519 (33.5%) were younger than 30 years, 770 (49.6%) were aged 31 to 35 years, 169 (10.9%) were aged 36 to 40 years, and 53 (3.4%) were older than 40 years. Among 1402 respondents indicating their parental status, 365 (26.0%) reported having children. Among 1457 respondents indicating their race and ethnicity, 310 (21.3%) were Asian, 67 (4.6%) were Black or African American, 83 (5.7%) were Hispanic or Latino, 54 (3.7%) were Middle Eastern or North African, 654 (44.9%) were White, 136 (9.3%) were of other race or ethnicity, and 153 (10.5%) preferred not to answer.

 eTable 1 in Supplement 1 provides additional descriptive characteristics of the sample. Participant characteristics differed by site but were broadly similar to those of the full eligible trainee population at each site. In addition, demographic proportions for the subset of participants represented in multivariable models uniformly differed by less than 1% from proportions for each variable’s individual respondents. Among the 1656 responders to the survey, 1491 (90.0%) completed the primary FI items and 1334 (80.6%) had complete data for independent variables and therefore were included in multivariable analysis with FI as a dependent variable. Missing data proportions for demographic variables are reported in eTable 1 in Supplement 1 and ranged from 0.5% for specialty to 15.3% for parental status.

### Prevalence of FI

The overall prevalence of FI was 13.7% (204 of 1491). In multivariable analyses (Table 1), there were differences in prevalence of FI across training sites, with a prevalence of 15.6%, 17.0%, and 21.3% at the 3 large metropolitan training sites compared with 4.5% at the small metropolitan site (*P* < .001). FI differed by trainee race and ethnicity as well; most notably, 22.4% of Black or African American residents had FI compared with 8.4% of White residents (*P* = .04). Postgraduate year 1 trainees had an FI prevalence of 16.9% compared with 10.1% for trainees in postgraduate year 5 or higher (*P* = .003). There were no statistically significant differences in FI by gender, sexual orientation, parental status, or specialty overall.

**Table 1.** Factors Associated With Food Insecurity Among Residents and Fellows^a^

Independent variable	Residents and fellows with food insecurity, No./total No. (%)	ARR (95% CI)	*P* value	Overall *P* value
Age, y				
<30	60/496 (12.1)	1 [Reference]	NA	.09
31-35	101/737 (13.7)	1.60 (1.10-2.33)	.01	
36-40	21/160 (13.1)	1.49 (0.84-2.64)	.18	
>40	6/50 (12.0)	1.91 (0.83-4.39)	.13	
Postgraduate year				
1	35/207 (16.9)	1 [Reference]	NA	.003
2	31/254 (12.2)	0.74 (0.48-1.17)	.20	
3	34/241 (14.1)	0.68 (0.42-1.08)	.11	
4	26/226 (11.5)	0.41 (0.24-0.71)	.002	
≥5	49/485 (10.1)	0.37 (0.22-0.62)	<.001	
Race and ethnicity				
Asian	45/308 (14.6)	1.46 (1.00-2.13)	.05	.04
Black or African American	15/67 (22.4)	2.35 (1.43-3.88)	<.001	
Hispanic or Latino	14/83 (16.9)	1.41 (0.76-2.62)	.28	
Middle Eastern or North African	10/54 (18.5)	2.34 (1.22-4.51)	.01	
White	55/654 (8.4)	1 [Reference]	NA	
Other^b^	20/136 (14.7)	1.17 (0.67-2.04)	.58	
Prefer not to answer	38/153 (24.8)	2.36 (1.32-4.22)	.004	
Site				
A	17/109 (15.6)	3.56 (2.01-6.33)	<.001	<.001
B	15/88 (17.0)	3.64 (2.03-6.50)	<.001	
C	28/622 (4.5)	1 [Reference]	NA	
D	144/676 (21.3)	3.95 (2.53-6.17)	<.001	
Gender				
Man	98/731 (13.4)	1 [Reference]	NA	.21
Woman	63/600 (10.5)	0.89 (0.65-1.21)	.46	
Other	5/12 (41.7)	2.27 (0.86-6.02)	.10	
Prefer not to answer	29/109 (26.6)	0.53 (0.22-1.28)	.16	
Sexual orientation				
Heterosexual	135/1184 (11.4)	1 [Reference]	NA	.58
Gay or lesbian	14/70 (20.0)	1.23 (0.69-2.19)	.48	
Bisexual	9/56 (16.1)	1.26 (0.64-2.49)	.50	
More than 1, not listed, or other	2/13 (15.4)	0.72 (0.12-4.24)	.71	
Prefer not to answer	37/130 (28.5)	1.66 (0.94-2.92)	.08	
Have children				
No	131/1031 (12.7)	1 [Reference]	NA	.29
Yes	45/363 (12.4)	1.22 (0.86-1.73)	.29	
Specialty (vs internal medicine)				
Anesthesia and perioperative care	14/90 (15.6)	1.26 (0.67-2.37)	.47	.12
Dermatology	7/45 (15.6)	1.41 (0.63-3.19)	.41	
Emergency medicine	12/53 (22.6)	1.17 (0.55-2.49)	.68	
Family and community medicine	8/58 (13.8)	1.50 (0.72-3.14)	.28	
Internal medicine	48/466 (10.3)	1 [Reference]	NA	
Laboratory medicine	1/30 (3.3)	0.58 (0.07-4.53)	.60	
Neurology	13/98 (13.3)	1.13 (0.56-2.29)	.74	
Obstetrics and gynecology	6/44 (13.6)	1.16 (0.52-2.61)	.72	
Ophthalmology	2/17 (11.8)	1.30 (0.22-7.76)	.77	
Orthopedic surgery	4/31 (12.9)	2.61 (1.15-5.94)	.02	
Other	1/9 (11.1)	0.25 (0.04-1.70)	.16	
Otolaryngology and head and neck surgery	4/41 (9.8)	0.86 (0.33-2.24)	.76	
Pathology	5/26 (19.2)	1.11 (0.42-2.88)	.84	
Pediatrics	26/132 (19.7)	1.15 (0.68-1.95)	.60	
Psychiatry and behavioral sciences	7/77 (9.1)	0.78 (0.34-1.81)	.56	
Radiology and diagnostic imaging	10/77 (13.0)	0.99 (0.46-2.11)	.98	
Surgery	27/122 (22.1)	2.40 (1.48-3.87)	<.001	
Urology	7/30 (23.3)	2.05 (1.00-4.20)	.05	

Abbreviations: ARR, adjusted relative risk; NA, not applicable.

^a^ Results exclude missing data for each variable. Missing data proportions are reported in eTable 1 in Supplement 1.

^b^ Includes American Indian or Alaska Native, Native Hawaiian or Other Pacific Islander, multiracial, and preferred response not listed but otherwise specified by the respondent.

### FI and Burnout

The prevalence of overall burnout in the sample was 41.6% (642 of 1545). In multivariable analysis, residents and fellows with FI were more likely to experience burnout (adjusted relative risk, 1.37 [95% CI, 1.18-1.60]; *P* < .001) (eTable 2 in Supplement 1), with a prevalence of burnout of 61.8% (126 of 204) for those with FI and 38.7% (497 of 1285) for those without FI (Figure). Other variables associated with burnout are detailed in eTable 2 in Supplement 1.

**Figure.**
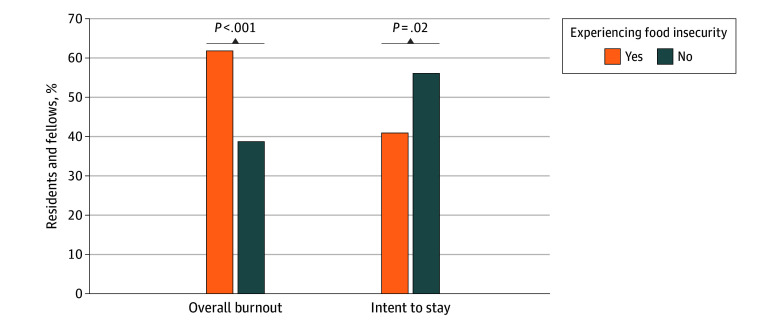
Burnout and Intent to Stay at Institution After Training for Residents and Fellows With and Without Food Insecurity

### FI and Intent to Stay at Institution

In multivariable analyses, residents with FI were less likely to consider remaining at their institution for further training or a faculty position (adjusted relative risk, 0.81 [95% CI, 0.68-0.98]; *P* = .02) (eTable 3 in Supplement 1), with 40.9% (83 of 203) of those with FI saying that they would consider staying at their institution compared with 56.1% (716 of 1277) of those without FI (Figure). Other variables associated with intent to stay are detailed in eTable 3 in Supplement 1.

### FI and Social Isolation

FI was also associated with higher social isolation scores (T-score parameter estimate, 2.37 [95% CI, 0.89-3.86]; *P* = .002) (eTable 4 in Supplement 1). Other variables associated with higher social isolation scores are detailed in eTable 4 in Supplement 1.

## Discussion

We report results from what is to our knowledge the first multisite study characterizing FI in resident and fellow physicians. We found that a substantial proportion of resident and fellow physicians, approximately 1 in 7, screened positive for FI. FI proportions differed across training sites, with higher levels at the 3 sites in large metropolitan locations, and trainees in postgraduate year 1 were more likely to have FI than those later in training. Consistent with prior studies, FI differed by race and ethnicity; the prevalence of FI was particularly high among Black or African American trainees. We also found that FI was significantly associated with burnout, social isolation, and lower intent to stay at one’s institution for further training or a faculty position.

The higher prevalence of FI in large metropolitan areas seen in this study comports with data from the general population showing higher risk for FI in major urban areas compared with suburban areas.^2^ It is not clear from our data whether this finding is due entirely to increased cost of living for the geographic location or is also related to indirect expenses specific to medical training. Despite being at or above the US average, resident and fellow salaries, particularly at lower postgraduate years, have been shown to be potentially insufficient for a living wage depending on factors such as geographic region, household size, and living expenses such as food, housing, and transportation costs.^16,17,18,33^ One study suggested that the resident stipend lost as much as half of its value in large metropolitan cities once adjusting for cost of living.^16^ Additionally, even in urban areas with lower overall cost of living, residents and fellows typically need to live close to the hospital; zip codes that contain large hospitals (typical of teaching hospitals) have been shown to have higher housing costs than those with small hospitals or without hospitals.^34^

In addition to location-related FI concerns, the racial and ethnic differences in FI and the higher risk experienced by trainees in postgraduate year 1 suggest that an interplay of demographic and financial factors is likely relevant to FI and its associations with burnout, social isolation, and intent to stay at one’s institution after training. The markedly higher prevalence of FI among Black or African American trainees in our study aligns with previous studies in which medical students of a racial or ethnic group that is underrepresented in medicine had nearly twice the prevalence of FI^9^ and Black or African American students were nearly 3 times as likely to experience FI compared with White students.^8^ Studies in medical students have also demonstrated higher financial stress among students who are the first in their family to attend college,^19,20^ as well as higher risk of FI among students from low-income families.^9^ Although we did not directly ask whether residents came from low-income families or were the first generation to attend college, postgraduate year has been shown to correlate directly with salary for residents,^17^ and postgraduate year 1 trainees who do not have a financial safety net may be particularly vulnerable to FI.

The increased risk for burnout among residents and fellows experiencing FI may further relate to additional stress due to lack of resources to meet basic needs on top of the high demands of residency or fellowship. For those experiencing FI, for example, social isolation and loneliness may result from an effort to avoid social events that require spending money, potentially compounding a lack of belonging or community.^12,13^ These factors may also contribute to the finding in this study that residents and fellows experiencing FI were less likely to report intention to stay at their institution.

Since residents and fellows eat many of their meals at work, risk for FI at a given institution could differ depending on the extent to which that institution subsidizes meal costs. Trainees at the sites in this study experienced FI despite each institution having food allowances for residents and fellows, and even with the same food allowance, FI levels differed markedly across sites within the same institution. This finding suggests that current food allowances or access may be insufficient or that additional solutions are required. Given the risk for FI identified in this study among residents and fellow physicians, and associated stigma related to FI, solutions which are broad-based are likely to be most impactful, despite some demographic groups being at higher risk. Residents and fellow physicians are generally not eligible for additional support programs such as Supplemental Nutritional Assistance Program benefits. Additionally, although the Accreditation Council for Graduate Medical Education currently requires that institutions provide access to food, there is no specific financial parameter for this and no requirement for sponsoring institutions to provide free or low-cost food.^35^ While the overall cost of living is not within an institution’s control, and many health system employees are at risk for FI, we contend that each institution has a particular responsibility to mitigate FI risk for its residents and fellows, who are contractually obligated to relocate and stay for training, spend a disproportionate number of hours and mealtimes at work, and provide an essential backbone of patient care at their sites.

Interventions can target FI among residents and fellows either by directly decreasing the proportion of income spent on food or by reducing other costs that limit available income to spend on food. As described in Table 2, institutions could, for example, expand free or low-cost food access on-site (including outside of typical mealtimes to account for the 24/7 nature of resident work). In 1 study, medical students perceived that they had lower FI during rotations that provided meals.^13^ In addition, institutions could ensure that salaries are commensurate with local living expenses, provide additional benefits to increase available income for food, and build mechanisms to support groups that may be at higher risk for FI. Examples of specific interventions that institutions may consider are listed in Table 2. Partnerships between trainees and national bodies such as the Accreditation Council for Graduate Medical Education might best ensure the development and implementation of effective and sustainable solutions.

**Table 2.** Potential Interventions to Address Resident and Fellow Food Insecurity

Target	Example interventions
Food costs	Free or reduced-cost food available at hospital and/or clinical sites, including outside of regular mealtimes
	Partnerships with local establishments for discounts
	Disseminating information about local options for lower-cost meals and groceries
Overall cost of living	Salary commensurate with local cost of living and housing costs
	Benefits that reduce other costs of living (health care and/or childcare flexible spending account, commuter benefits account)
	Moving allowance
Targeted interventions for at-risk groups	Salary advance or interest-free loan for residents and/or fellows transitioning into training
	Availability of food bags and resources at events likely to have a high proportion at risk for food insecurity (postgraduate year 1 orientation, first generation to attend college)

### Limitations

This study has several limitations. First, the data are cross-sectional, therefore direction of association cannot be definitively determined. However, while FI results from a complex interplay of drivers, it is more plausible that burnout results from rather than causes FI. Second, while many of our findings align with known risk factors for FI, the study included limited demographic information, restricting our ability to further examine drivers of FI such as lack of a financial safety net or caring for family members other than children. Our study was not designed to elicit specific reasons why trainees did not want to remain at their institution, and it is possible that factors other than those we adjusted for contributed to this outcome. In addition, other factors correlated with urbanicity of a training location might account for observed differences in FI across training sites and should be explored in studies that include additional locations. While this study included 4 training sites at 2 academic medical institutions, experiences at other institutions may differ. Finally, the survey response rate leaves open the possibility of response bias in estimating the true prevalence of FI; the similarity of respondents to the eligible trainee populations at participating sites is reassuring but not conclusive in this regard.

## Conclusions

In this cross-sectional study of resident and fellow physicians at 4 geographically distinct training sites across 2 academic institutions, nearly 1 in 7 screened positive for FI, especially at large metropolitan training sites. FI in residents and fellows was associated with important well-being outcomes including burnout. Institutions should include attention to systemic solutions to address FI in residents and fellows as a critical component of supporting resident and fellow well-being.

## Notes

Supplement 1. **eTable 1.** Sample Demographics Overall and by Site **eTable 2.** Factors Associated With Burnout in Residents and Fellows **eTable 3.** Factors Associated With Intent to Stay at Institution for Further Training or Faculty Position in Residents and Fellows **eTable 4.** Factors Associated With Social Isolation in Residents and Fellows

Supplement 2.
Data Sharing Statement
