# Supplementary material for: Food Insecurity, Burnout, and Social Isolation Among Resident and Fellow Physicians
Source: JAMA Netw Open. 2025 Dec 17;8(12):e2550044. doi: 10.1001/jamanetworkopen.2025.50044 (PMC12712728; doi:10.1001/jamanetworkopen.2025.50044)
Supplement: Supplement 1. — eTable 1. Sample Demographics Overall and by Site eTable 2. Factors Associated With Burnout in Residents and Fellows eTable 3. Factors Associated With Intent to Stay at Institution for Further Training or Faculty Position in Residents and Fellows eTable 4. Factors Associated With Social Isolation in Residents and Fellows [file jamanetwopen-e2550044-s001.pdf]

Supplemental Online Content

Thomas LR, Dyrbye LN, Satele D, et al. Food insecurity, burnout, and social isolation among resident and fellow physicians. *JAMA Netw Open*. 2025;8(12):e2550044. doi:10.1001/jamanetworkopen.2025.50044

eTable 1. Sample Demographics Overall and by Site
eTable 2. Factors Associated With Burnout in Residents and Fellows
eTable 3. Factors Associated With Intent to Stay at Institution for Further Training or Faculty Position in Residents and Fellows
eTable 4. Factors Associated With Social Isolation in Residents and Fellows

This supplemental material has been provided by the authors to give readers additional information about their work.

© 2025 Thomas LR et al. *JAMA Network Open*.

eTable 1. Sample Demographics Overall and by Site

		No. (%) Within Site[a]				
	Total (N=1656)	A (N=117)	B (N=99)	C (N=664)	D (N=776)	*P*-value[a,b]
**Age category**						<.001
20-25	2 (0.1%)	0 (0.0%)	0 (0.0%)	2 (0.3%)	0 (0.0%)	
26-30	517 (33.3%)	51 (43.6%)	33 (33.3%)	245 (36.9%)	188 (28.0%)	
31-35	770 (49.6%)	47 (40.2%)	48 (48.5%)	317 (47.7%)	358 (53.4%)	
36-40	169 (10.9%)	14 (12.0%)	12 (12.1%)	71 (10.7%)	72 (10.7%)	
>40	53 (3.4%)	5 (4.3%)	6 (6.1%)	29 (4.4%)	13 (1.9%)	
Prefer not to answer	40 (2.6%)	0 (0.0%)	0 (0.0%)	0 (0.0%)	40 (6.0%)	
Missing	105	0	0	0	105	
**Gender identity**						<.001
Woman	602 (41.3%)	42 (39.3%)	37 (42.5%)	302 (50.0%)	221 (33.5%)	
Man	735 (50.4%)	60 (56.1%)	47 (54.0%)	275 (45.5%)	353 (53.5%)	
Other	12 (0.8%)	1 (0.9%)	0 (0.0%)	5 (0.8%)	6 (0.9%)	
Prefer not to answer	109 (7.5%)	4 (3.7%)	3 (3.4%)	22 (3.6%)	80 (12.1%)	
Missing	198	10	12	60	116	
**Race/ethnicity**						<.001
Asian	310 (21.3%)	21 (19.3%)	18 (20.7%)	91 (15.1%)	180 (27.4%)	
African American/Black	67 (4.6%)	5 (4.6%)	2 (2.3%)	24 (4.0%)	36 (5.5%)	
Latino/Hispanic	83 (5.7%)	2 (1.8%)	3 (3.4%)	25 (4.1%)	53 (8.1%)	
Middle Eastern/North African	54 (3.7%)	2 (1.8%)	7 (8.0%)	28 (4.6%)	17 (2.6%)	
White	654 (44.9%)	59 (54.1%)	39 (44.8%)	340 (56.3%)	216 (32.9%)	
Other or More than one race[c]	136 (9.3%)	13 (11.9%)	6 (6.9%)	49 (8.1%)	68 (10.4%)	
Prefer not to answer	153 (10.5%)	7 (6.4%)	12 (13.8%)	47 (7.8%)	87 (13.2%)	
Missing	199	8	12	60	119	
**Sexual orientation**						<.001
Bisexual	56 (3.8%)	2 (1.8%)	0 (0.0%)	24 (4.0%)	30 (4.6%)	
Gay/lesbian	70 (4.8%)	5 (4.6%)	2 (2.4%)	14 (2.3%)	49 (7.4%)	
Heterosexual	1186 (81.5%)	98 (89.9%)	78 (91.8%)	533 (88.5%)	477 (72.4%)	
More than one, not listed/other	13 (0.9%)	0 (0.0%)	0 (0.0%)	6 (1.0%)	7 (1.1%)	
Prefer not to answer	130 (8.9%)	4 (3.7%)	5 (5.9%)	25 (4.2%)	96 (14.6%)	
Missing	201	8	14	62	117	
**Do you have any children?**						<.001
Yes	365 (26.0%)	39 (35.8%)	38 (43.7%)	180 (29.8%)	108 (18.0%)	
No	1037 (74.0%)	70 (64.2%)	49 (56.3%)	425 (70.2%)	493 (82.0%)	
Missing	254	8	12	59	175	
**Post Graduate Year**						0.26
1	222 (15.0%)	22 (18.8%)	15 (15.2%)	107 (16.1%)	78 (13.0%)	

© 2025 Thomas LR et al. *JAMA Network Open*.

2	263 (17.7%)	22 (18.8%)	23 (23.2%)	121 (18.2%)	97 (16.1%)	
3	252 (17.0%)	21 (17.9%)	14 (14.1%)	118 (17.8%)	99 (16.4%)	
4	240 (16.2%)	18 (15.4%)	17 (17.2%)	90 (13.6%)	115 (19.1%)	
5+	505 (34.1%)	34 (29.1%)	30 (30.3%)	228 (34.3%)	213 (35.4%)	
Missing	174	0	0	0	174	
**Department**						<.001
Anesthesia & Perioperative Care	98 (5.9%)	9 (7.7%)	7 (7.1%)	40 (6.0%)	42 (5.5%)	
Dentistry	7 (0.4%)	0 (0.0%)	0 (0.0%)	7 (1.1%)	0 (0.0%)	
Dermatology	48 (2.9%)	4 (3.4%)	2 (2.0%)	17 (2.6%)	25 (3.3%)	
Emergency Medicine	58 (3.5%)	0 (0.0%)	0 (0.0%)	16 (2.4%)	42 (5.5%)	
Family and Community Medicine	64 (3.9%)	0 (0.0%)	9 (9.1%)	39 (5.9%)	16 (2.1%)	
Internal Medicine	517 (31.4%)	40 (34.2%)	46 (46.5%)	230 (34.6%)	201 (26.2%)	
Laboratory Medicine	34 (2.1%)	1 (0.9%)	1 (1.0%)	29 (4.4%)	3 (0.4%)	
Neurological Surgery	20 (1.2%)	1 (0.9%)	2 (2.0%)	11 (1.7%)	6 (0.8%)	
Neurology	104 (6.3%)	7 (6.0%)	10 (10.1%)	37 (5.6%)	50 (6.5%)	
Obstetrics & Gynecology	50 (3.0%)	2 (1.7%)	1 (1.0%)	17 (2.6%)	30 (3.9%)	
Ophthalmology	22 (1.3%)	0 (0.0%)	1 (1.0%)	6 (0.9%)	15 (2.0%)	
Orthopaedic Surgery	38 (2.3%)	0 (0.0%)	0 (0.0%)	27 (4.1%)	11 (1.4%)	
Other	9 (0.5%)	6 (5.1%)	0 (0.0%)	3 (0.5%)	0 (0.0%)	
Otolaryngology and Head and Neck Surgery	43 (2.6%)	5 (4.3%)	0 (0.0%)	12 (1.8%)	26 (3.4%)	
Pathology	27 (1.6%)	0 (0.0%)	0 (0.0%)	0 (0.0%)	27 (3.5%)	
Pediatrics	143 (8.7%)	0 (0.0%)	1 (1.0%)	28 (4.2%)	114 (14.8%)	
Psychiatry and Behavioral Sciences	83 (5.0%)	0 (0.0%)	1 (1.0%)	37 (5.6%)	45 (5.9%)	
Radiation Oncology	25 (1.5%)	2 (1.7%)	0 (0.0%)	13 (2.0%)	10 (1.3%)	
Radiation and Diagnostic Imaging	91 (5.5%)	14 (12.0%)	10 (10.1%)	25 (3.8%)	42 (5.5%)	
Surgery	133 (8.1%)	21 (17.9%)	7 (7.1%)	58 (8.7%)	47 (6.1%)	
Urology	34 (2.1%)	5 (4.3%)	1 (1.0%)	12 (1.8%)	16 (2.1%)	
Missing	8	0	0	0	8	

[a]Results exclude missing data for each variable
[b]Chi-Square p-value
[c]"Other" includes Native American or Alaskan Native, Native Hawaiian or Other Pacific Islander, and Preferred response not listed but otherwise specified by respondents.

© 2025 Thomas LR et al. *JAMA Network Open*.

eTable 2. Factors Associated With Burnout in Residents and Fellows

Independent Variable	aRR (95% CI)	*P*-value	Overall *P*-value[a]
**Food Insecurity, Positive Screen**	1.37 (1.18-1.60)	--	<.001
**Age (vs. <30 years)**			.02
31-35	1.06 (0.91-1.24)	.42	
36-40	0.80 (0.60-1.07)	.14	
>40	0.59 (0.33-1.06)	.08	
**Postgraduate Year (vs. 1)**			<.001
2	1.30 (1.06-1.60)	.01	
3	1.32 (1.07-1.62)	.009	
4	0.89 (0.69-1.16)	.38	
5+	1.00 (0.80-1.26)	.97	
**Sexual Orientation (vs. Heterosexual)**			.03
Gay/lesbian	1.20 (0.96-1.50)	.11	
Bisexual	1.24 (0.99-1.55)	.07	
More than one, not listed/other	1.97 (1.41-2.77)	<.001	
Prefer not to answer	1.20 (0.91-1.58)	.20	
**Have Children**	0.77 (0.64-0.93)	--	.006
**Site (vs. C)**			<.001
A	1.42 (1.09-1.84)	.009	
B	1.10 (0.80-1.52)	.56	
D	1.73 (1.47-2.03)	<.001	
**Specialty (vs. Internal Medicine)**			.002
Anesthesia & Perioperative Care	1.30 (1.01-1.69)	.04	
Dermatology	1.04 (0.70-1.54)	.85	
Emergency Medicine	0.94 (0.68-1.30)	.70	
Family and Community Medicine	1.34 (0.99-1.80)	.06	
Laboratory Medicine	0.57 (0.23-1.39)	.22	
Neurology	1.02 (0.76-1.37)	.91	
Obstetrics & Gynecology	1.32 (0.96-1.82)	.09	
Ophthalmology	1.89 (1.30-2.75)	<.001	
Orthopedic Surgery	0.44 (0.20-0.96)	.04	
Other	1.17 (0.81-1.68)	.41	
Otolaryngology and Head and Neck Surgery	0.80 (0.50-1.28)	.34	
Pathology	0.85 (0.51-1.42)	.54	
Pediatrics	1.13 (0.90-1.41)	.29	
Psychiatry and Behavioral Sciences	1.20 (0.92-1.55)	.17	

© 2025 Thomas LR et al. *JAMA Network Open*.

Radiology and Diagnostic Imaging	0.73 (0.48-1.09)	.12	
Surgery	1.24 (0.97-1.58)	.08	
Urology	1.26 (0.88-1.78)	.20	
**Gender (vs. Man)**			.04
Woman	1.21 (1.06-1.39)	.004	
Other	0.97 (0.46-2.03)	.93	
Prefer not to answer	1.09 (0.71-1.68)	.70	
**Race/Ethnicity (vs. White)**			.04
African American/Black	0.82 (0.62-1.09)	.18	
Asian	0.91 (0.76-1.07)	.26	
Latino/Hispanic	0.80 (0.60-1.07)	.13	
Middle Eastern/North African	0.64 (0.40-1.05)	.08	
Other or More than one race[b]	0.88 (0.70-1.10)	.26	
Prefer not to answer	1.30 (0.94-2.07)	.10	

[a]Results exclude missing data for each variable.
[b]"Other" includes Native American or Alaskan Native, Native Hawaiian or Other Pacific Islander, and Preferred response not listed but otherwise specified by respondents.

© 2025 Thomas LR et al. *JAMA Network Open*.

eTable 3. Factors Associated With Intent to Stay at Institution for Further Training or Faculty Position in Residents and Fellows

Independent Variables	aRR (95% CI)	*P*-value	Overall *P*-value[a]
**Food Insecurity, Positive Screen**	0.81 (0.68-0.98)	--	.02
**Race/Ethnicity (vs. White)**			.03
African American/Black	0.94 (0.76-1.18)	.61	
Asian	0.90 (0.79-1.02)	.10	
Latino/Hispanic	1.19 (0.99-1.42)	.06	
Middle Eastern/North African	0.87 (0.68-1.11)	.26	
Other or More than one race[b]	0.92 (0.77-1.10)	.36	
Prefer not to answer	0.72 (0.55-0.95)	.02	
**Have Children**	1.14 (1.02-1.27)	--	.02
**Site (vs. C)**			<.001
A	0.86 (0.72-1.03)	.11	
B	1.06 (0.90-1.26)	.50	
D	0.80 (0.71-0.90)	<.001	
**Specialty (vs. Internal Medicine)**			.01
Anesthesia & Perioperative Care	0.61 (0.46-0.81)	<.001	
Dermatology	0.91 (0.67-1.25)	.57	
Emergency Medicine	0.90 (0.66-1.22)	.50	
Family and Community Medicine	0.92 (0.73-1.15)	.46	
Laboratory Medicine	0.89 (0.64-1.25)	.51	
Neurology	1.00 (0.83-1.20)	.96	
Obstetrics & Gynecology	0.71 (0.49-1.04)	.08	
Ophthalmology	0.17 (0.03-1.04)	.08	
Orthopedic Surgery	0.91 (0.68-1.22)	.53	
Other	1.00 (0.77-1.29)	.99	
Otolaryngology and Head and Neck Surgery	1.14 (0.88-1.47)	.31	
Pathology	1.20 (0.84-1.74)	.32	
Pediatrics	0.99 (0.82-1.20)	.90	
Psychiatry and Behavioral Sciences	0.86 (0.67-1.10)	.22	
Radiology and Diagnostic Imaging	1.06 (0.87-1.30)	.54	
Surgery	1.00 (0.84-1.18)	.95	
Urology	1.09 (0.80-1.48)	.58	
**Age (vs. <30 years)**			.11
31-35	0.97 (0.86-1.10)	.62	
36-40	1.15 (0.97-1.36)	.10	
>40	1.13 (0.88-1.46)	.34	
**Gender (vs. Man)**			.50

© 2025 Thomas LR et al. *JAMA Network Open*.

Woman	0.93 (0.85-1.02)	.14	
Other	0.95 (0.50-1.84)	.89	
Prefer not to answer	0.89 (0.58-1.37)	.60	
**Sexual Orientation (vs. Heterosexual)**			
Gay/lesbian	0.88 (0.66-1.17)	.38	
Bisexual	0.95 (0.74-1.21)	.66	.23
More than one, not listed/other	0.43 (0.16-1.14)	.09	
Prefer not to answer	1.05 (0.77-1.42)	.78	
**Postgraduate Year (vs. 1)**			
2	0.83 (0.70-0.97)	.02	
3	0.97 (0.83-1.13)	.69	.07
4	0.92 (0.78-1.10)	.36	
5+	0.85 (0.73-1.00)	.049	

[a] Results exclude missing data for each variable.
[b]"Other" includes Native American or Alaskan Native, Native Hawaiian or Other Pacific Islander, and Preferred response not listed but otherwise specified by respondents.

© 2025 Thomas LR et al. *JAMA Network Open*.

eTable 4. Factors Associated With Social Isolation in Residents and Fellows

Independent Variable	Adjusted Parameter Estimate (95% CI)	*P*-value	Overall *P*-value[a]
**Food Insecurity, Positive Screen**	2.37 (0.89-3.86)	--	.002
**Postgraduate Year (vs. 1)**			.01
2	0.94 (-0.72-2.59)	.27	
3	-0.51 (-2.19-1.18)	.56	
4	-1.82 (-3.63-(-0.01))	.049	
5+	-1.44 (-3.12-0.24)	.09	
**Sexual Orientation (vs. Heterosexual)**			.02
Gay/lesbian	2.08 (-0.11-4.25)	.06	
Bisexual	2.17 (-0.21-4.55)	.07	
More than one, not listed/other	5.22 (0.30-10.13)	.04	
Prefer not to answer	2.08 (-0.78-4.93)	.15	
**Site (vs. C)**			.01
A	-1.10 (-2.91-0.71)	.23	
B	-0.45 (-2.43-1.54)	.66	
D	1.50 (0.37-2.63)	.01	
**Specialty (vs. Internal Medicine)**			<.001
Anesthesia & Perioperative Care	3.93 (1.90-5.97)	<.001	
Dermatology	-0.56 (-3.44-2.33)	.71	
Emergency Medicine	0.03 (-2.65-2.70)	.99	
Family and Community Medicine	-1.99 (-4.46-0.47)	.11	
Laboratory Medicine	-0.92 (-4.27-2.43)	.59	
Neurology	-0.90 (-2.90-1.09)	.38	
Obstetrics & Gynecology	1.75 (-1.06-4.57)	.22	
Ophthalmology	-1.45 (-6.78-3.88)	.59	
Orthopedic Surgery	-4.53 (-7.77-(-1.29))	.006	
Other	-0.67 (-3.25-1.91)	.61	
Otolaryngology and Head and Neck Surgery	-4.37 (-7.17-(-1.56))	.002	
Pathology	3.15 (-0.59-6.88)	.10	
Pediatrics	-0.18 (-2.02-1.66)	.85	
Psychiatry and Behavioral Sciences	1.54 (-0.65-3.73)	.17	
Radiology and Diagnostic Imaging	-1.63 (-3.85-0.60)	.15	
Surgery	0.98 (-0.82-2.79)	.29	
Urology	-1.33 (-4.56-1.89)	.42	
**Age (vs. <30 years)**			.17
31-35	1.06 (-0.15-2.26)	.09	
36-40	1.91 (0.08-3.74)	.04	
>40	0.50 (-2.31-3.31)	.73	

© 2025 Thomas LR et al. *JAMA Network Open*.

**Gender (vs. Man)**			.22
Woman	0.59 (-0.37-1.54)	.23	
Other	1.03 (-4.20-6.25)	.70	
Prefer not to answer	-2.92 (-6.61-0.77)	.12	
**Race/Ethnicity (vs. White)**			.12
African American/Black	0.58 (-1.60-2.75)	.60	
Asian	0.95 (-0.28-2.17)	.13	
Latino/Hispanic	0.39 (-1.67-2.44)	.71	
Middle Eastern/North African	0.79 (-1.65-3.23)	.53	
Other or More than one race[b]	-0.57 (-2.19-1.06)	.49	
Prefer not to answer	3.10 (0.81-5.39)	.008	
**Have Children**	-0.73 (-1.90-0.43)	--	.22

[a] Results exclude missing data for each variable.
[b]"Other" includes Native American or Alaskan Native, Native Hawaiian or Other Pacific Islander, and Preferred response not listed but otherwise specified by respondents.

© 2025 Thomas LR et al. *JAMA Network Open*.
