# Supplementary material for: Food Insecurity, Burnout, and Social Isolation Among Resident and Fellow Physicians
Source: JAMA Netw Open. 2025 Dec 17;8(12):e2550044. doi: 10.1001/jamanetworkopen.2025.50044 (PMC12712728; doi:10.1001/jamanetworkopen.2025.50044)
Supplement: Supplement 2. — Data Sharing Statement [file jamanetwopen-e2550044-s002.pdf]

# Data Sharing Statement

Thomas. Food Insecurity, Burnout, and Social Isolation Among Resident and Fellow Physicians. *JAMA Netw Open*. Published December 17, 2025.
doi:10.1001/jamanetworkopen.2025.50044

**Data**
**Data available:** No
